# CT based intratumor and peritumoral radiomics for differentiating complete from incomplete capsular characteristics of parotid pleomorphic adenoma: a two-center study

**DOI:** 10.1007/s12672-023-00665-8

**Published:** 2023-05-22

**Authors:** Shuang Li, Xiaorui Su, Youquan Ning, Simin Zhang, Hanbing Shao, Xinyue Wan, Qiaoyue Tan, Xibiao Yang, Juan Peng, Qiyong Gong, Qiang Yue

**Affiliations:** 1 Huaxi MR Research Center (HMRRC), Department of Radiology, West China Hospital of Sichuan University, Chengdu, 610041 China; 2 Research Unit of Psychoradiology, Chinese Academy of Medical Sciences, Chengdu, Sichuan China; 3 Functional and Molecular Imaging Key Laboratory of Sichuan Province, West China Hospital of Sichuan University, Chengdu, Sichuan China; 4 Department of Radiology, The First Affiliated Hospital of Chongqing Medical University, Chongqing, China; 5 Division of Radiation Physics, State Key Laboratory of Biotherapy and Cancer Center, West China Hospital of Sichuan University, Chengdu, China; 6 Department of Radiology, West China Hospital of Sichuan University, #37 GuoXue Xiang, Chengdu, 610041 Sichuan China; 7 Department of Radiology, West China Xiamen Hospital of Sichuan University, Xiamen, Fujian China

**Keywords:** Pleomorphic adenoma, Radiomics, Machine learning, Precise treatment

## Abstract

**Objective:**

Capsular characteristics of pleomorphic adenoma (PA) has various forms. Patients without complete capsule has a higher risk of recurrence than patients with complete capsule. We aimed to develop and validate CT-based intratumoral and peritumoral radiomics models to make a differential diagnosis between parotid PA with and without complete capsule.

**Methods:**

Data of 260 patients (166 patients with PA from institution 1 (training set) and 94 patients (test set) from institution 2) were retrospectively analyzed. Three Volume of interest (VOIs) were defined in the CT images of each patient: tumor volume of interest (VOI_tumor_), VOI_peritumor_, and VOI_intra-plus peritumor_. Radiomics features were extracted from each VOI and used to train nine different machine learning algorithms. Model performance was evaluated using receiver operating characteristic (ROC) curves and the area under the curve (AUC).

**Results:**

The results showed that the radiomics models based on features from VOI_intra-plus peritumor_ achieved higher AUCs compared to models based on features from VOI_tumor_. The best performing model was Linear discriminant analysis, which achieved an AUC of 0.86 in the tenfold cross-validation and 0.869 in the test set. The model was based on 15 features, including shape-based features and texture features.

**Conclusions:**

We demonstrated the feasibility of combining artificial intelligence with CT-based peritumoral radiomics features can be used to accurately predict capsular characteristics of parotid PA. This may assist in clinical decision-making by preoperative identification of capsular characteristics of parotid PA.

**Supplementary Information:**

The online version contains supplementary material available at 10.1007/s12672-023-00665-8.

## Introduction

Pleomorphic adenomas (PA) are the most common type of benign tumors that occur in the parotid gland, accounting for 50–60% of all parotid tumors [1–3]. These slow-growing tumors are generally considered low-risk. Despite their benign nature, however, PAs have a relatively high risk of recurrence and malignant transformation [4]. Previous studies reported that capsular characteristics and surgical factors are the most likely causes for recurrence [5, 6].

Capsular characteristics refer to the appearance of the outer layer or capsule surrounding a parotid pleomorphic adenoma. A well-defined capsule is desirable as it facilitates complete tumor removal during surgery, reducing the risk of recurrence. However, capsules may vary in appearance. For example, some capsules may be thin and delicate. In some cases, the capsule may also be irregular or indistinct, making it challenging to determine the true extent of the tumor and increasing the risk of incomplete removal [7–9]. Pseudopodia and satellite nodules can permeate through the surrounding parotid tissue and thus are likely to be left when the surgical margin is not enough [10]. Capsular characteristics have led to an evolution of surgical methods for parotid PA [11, 12]. The goal is to remove the tumor completely while preserving the surrounding normal tissue and minimizing the risk of recurrence.

Currently, there is no clinically applicable, non-invasive method for preoperative evaluation of capsular characteristics. Fine-needle aspiration cytology cannot evaluate the capsular characteristics. Besides, visual inspection of computed tomography (CT) images, even by practiced radiologists to evaluate the capsular characteristics of PA, showed poor consistency with pathology [13]. Radiomics, a medical imaging field that extracts and analyzes quantitative data from images [14]. Intratumoral radiomics is a promising tool for identifying different pathological types of parotid tumors, with the number of patients included in previous studies ranging from 101 to 127 [15–17]. Intratumoral radiomics focuses on characterizing the heterogeneity and complexity of the tumor itself and can provide information on the molecular and biological characteristics of the tumor. Peritumoral radiomics, on the other hand, focuses on the changes in the surrounding tissue and can be used to better understand the interactions between the tumor and its surrounding tissue [18].

In this study, we aimed to investigate the use of radiomics for preoperative evaluation of capsular characteristics to guide surgical plans and patient management. We hypothesized that peritumoral radiomics features, which reflect the changes of tissues around tumor, might provide valuable information for the possible infiltration of tumor towards normal tissue.

## Material and methods

### Patient cohort

We searched the medical data of patients with PA at two institutions between January 2012 and February 2022. To gather the data, we utilized an electronic search of the Picture Archiving and Communication System (PACS) and Hospital Information System (HIS). Our institutional review board approved the study and waived the requirement for obtaining informed consent from patients. De-identifying images protected the privacy of the patients before analysis.

The study included patients who met the following criteria: (1) they had a confirmed diagnosis of PA through surgical pathology; (2) they underwent a contrast-enhanced CT scan before receiving any treatment. The study excluded patients who: (1) were diagnosed with carcinoma ex pleomorphic adenoma; (2) had a history of recurrent PA or previous parotid gland surgery; (3) had images that were compromised by significant metallic artifacts. The selection process for the patients is depicted in the flow diagram in Additional file 1: Figure S1. In the end, a total of 260 patients were included in the study, with 166 patients from our institution serving as the training cohort (92 with a complete capsule and 74 without a complete capsule), and 94 patients from another institution serving as the test cohort (49 with a complete capsule and 45 without a complete capsule).

### The reference standard for capsular characteristics

In this study, we used the following nomenclature, which is consistent with previous research [8]:

1. “Incomplete capsule” refers to a partial absence of encapsulation.
2. “Capsule penetration” refers to tumor tissue that has infiltrated into the tumor capsule but is not separated from the main mass by fibrous fibers.
3. “Pseudopodia” refers to tumor nodules that are separated from the main tumor mass by fibrous tissue but remain confined to or in contact with the main tumor capsule.
4. “Satellite nodules” refers to nodules located adjacent to the main tumor but not connected to it, and are separated from the main tumor by salivary glands or fat tissue.

Patients with incomplete capsule, capsule penetration, pseudopodia, and satellite nodules were grouped together, and patients with a complete capsule were grouped separately.

All patients in this study had undergone complete resection. The diagnosis of PA with a complete capsule was based on both surgical and pathological reports written by surgeons and pathologists. They were assigned to this group when the surgeon confirmed that the lesion was well-defined, and the pathologist confirmed the capsular completeness based on the histological certainty of the entire circumference of the lesion. The diagnosis of PA without a complete capsule was made based on pathological reports, which included the assessment of its completeness as well as other capsular characteristics such as capsule penetration, pseudopodia, and satellite nodules.

### CT image acquisition

All patients underwent CT scans within a week prior to their surgery. The CT images were saved in the Digital Imaging and Communications in Medicine (DICOM) format. The CT details can be found in the Additional file 1.

### Tumor segmentation and definition of peritumor size

We used ITK-SNAP software (http://www.itksnap.org) to analyze preoperative contrast-enhanced CT images. The software includes semi-automatic delineation algorithms to aid in the process. Nevertheless, the final volume of interest (VOI) was confirmed by experienced radiologists to ensure accuracy by reviewing all the CT image slices. The VOI was determined by encompassing the entire tumor while excluding adjacent bone regions and blood vessels. Two radiologists, with 3 and 5 years of clinical experience, performed the segmentation of the VOIs without knowledge of the patient’s clinical and pathological details. To evaluate the reliability of radiomics feature extraction, we computed both intra- and inter-observer correlation coefficients. A month later, the same radiologist repeated the segmentation process. We only considered features with intra- and inter-observer correlation coefficients of at least 0.80 for further analysis.

**Fig. 1:**
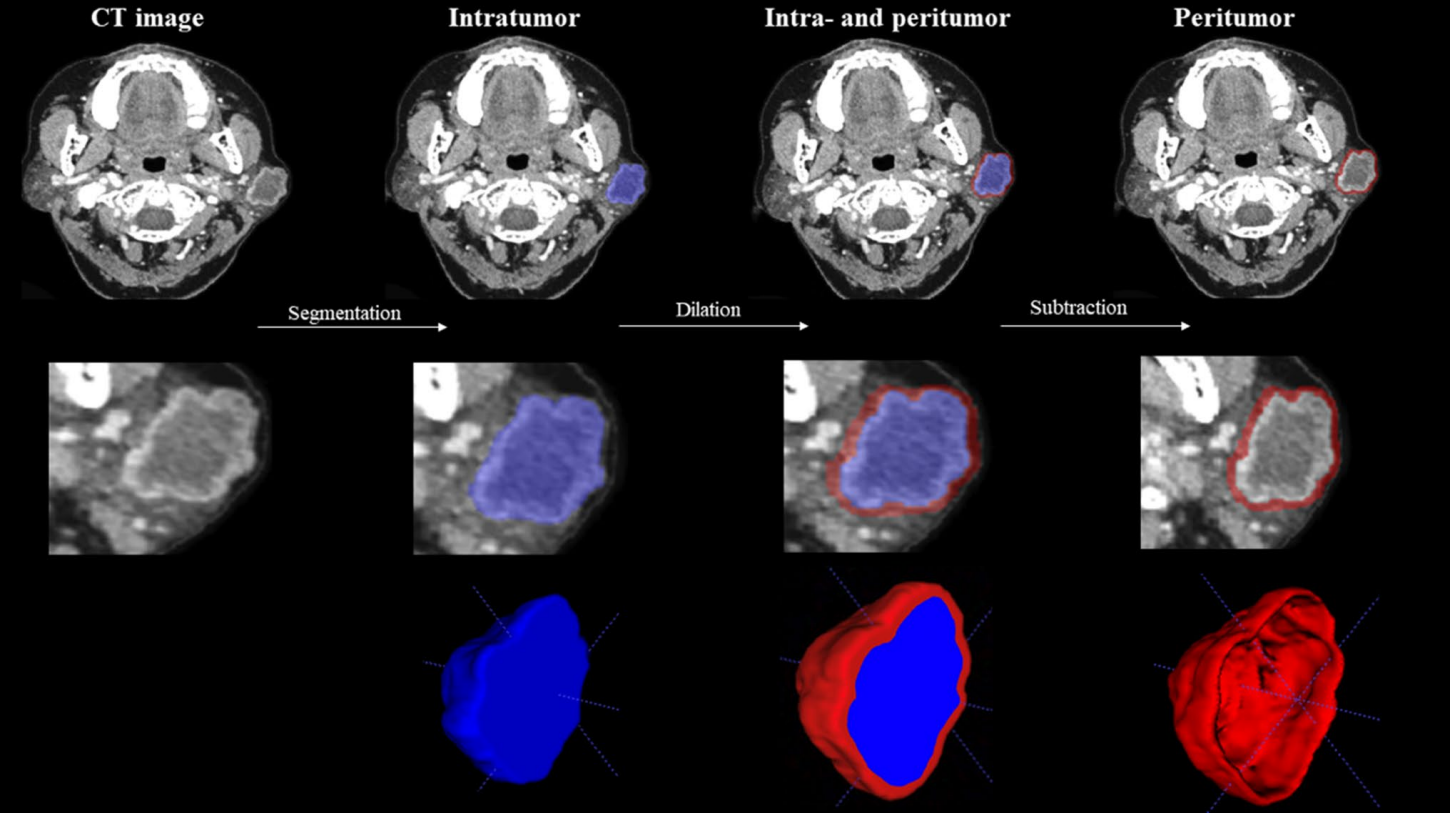
The process of obtaining three different regions

To generate the VOI intra-plus peritumor, we followed these steps:

We first identified the tumor’s VOI, which included the entire tumor while excluding adjacent bone regions and blood vessels. Next, we defined a 2 mm peritumoral region around the tumor based on its histological characteristics. This region captures the capsular characteristics typically found at the tumor’s margin and the satellite nodules predominantly located within 2 mm of the central mass [19]. Using the RIAS software package (version 0.1.2), we applied a morphological dilation operator to expand the intratumoral region by 2 mm in radial distance according to pixel size. This resulted in a new VOI that encompassed both the tumor and the peritumoral region, which we named “VOI_intra-plus peritumor_”. Finally, we subtracted the VOI_tumor_ from the VOI_intra-plus peritumor_ to obtain a circular VOI (VOI_peritumor_) using the same RIAS software package. Figure 1 depicts the process of dilation and subtraction.

In this axial view of an enhanced CT scan of a 63-year-old woman with an incomplete encapsulated pleomorphic adenoma of the parotid gland. The intratumoral mask is first obtained. The peritumoral region is then obtained by dilating the intratumoral mask. Finally, the ring of parotid parenchyma around the tumor is obtained by subtracting the original intratumoral mask from the dilated mask.

### Feature extraction

Before feature extraction, all CT images were resampled to a voxel size of 1 × 1 × 1 mm^3^ and normalized using the Z-score normalization method implemented in the RIAS software package (version 0.1.2). We utilized the PyRadiomics library (version 2.0.0) to extract features from segmented VOIs [20]. For each region, 18 first-order features, 22 Gy-level co-occurrence matrix (GLCM) features, 16 Gy level run length matrix (GLRLM) features, 16 Gy level size zone matrix (GLSZM) features, 14 Gy level dependence matrix (GLDM) features, 5 neighboring gray-tone difference matrix (NGTDM) features were obtained. Laplacian of Gaussian (LoG) convolution kernel filter (σ range from 1 to 5 mm, in incremental steps of 1 mm) to minimize noise and enhance features at different spatial scales [21]. To ensure that each region has the same number of features, tumor shape features are added to each region. Each region has 1288 radiomics features: 14 shape features extracted from tumor + 91 textural features × (1 original image + 5 LoG filtered images + 8 wavelet filtered images). A detailed list of extracted features is provided in Additional file 1: Table S1 and the parameters used in CT image pre-processing and feature extraction is provided in Additional file 1: Table S2.

### Radiomics features pre-processing and selection

The dataset was normalized through Z-score normalization, which involved subtracting the mean value and dividing by the standard deviation for each feature. We reduced the dimensionality of the feature space by comparing the similarity of each feature pair and removing one of the features if the Pearson correlation coefficient (PCC) value was higher than 0.99. We applied the Kruskal Wallis (KW) test to identify significant features related to the labels and calculated the F-value to assess the relationship between the features and the label. The number of features ranged from 1 to 20.

### Model building and validation

**Fig. 2:**
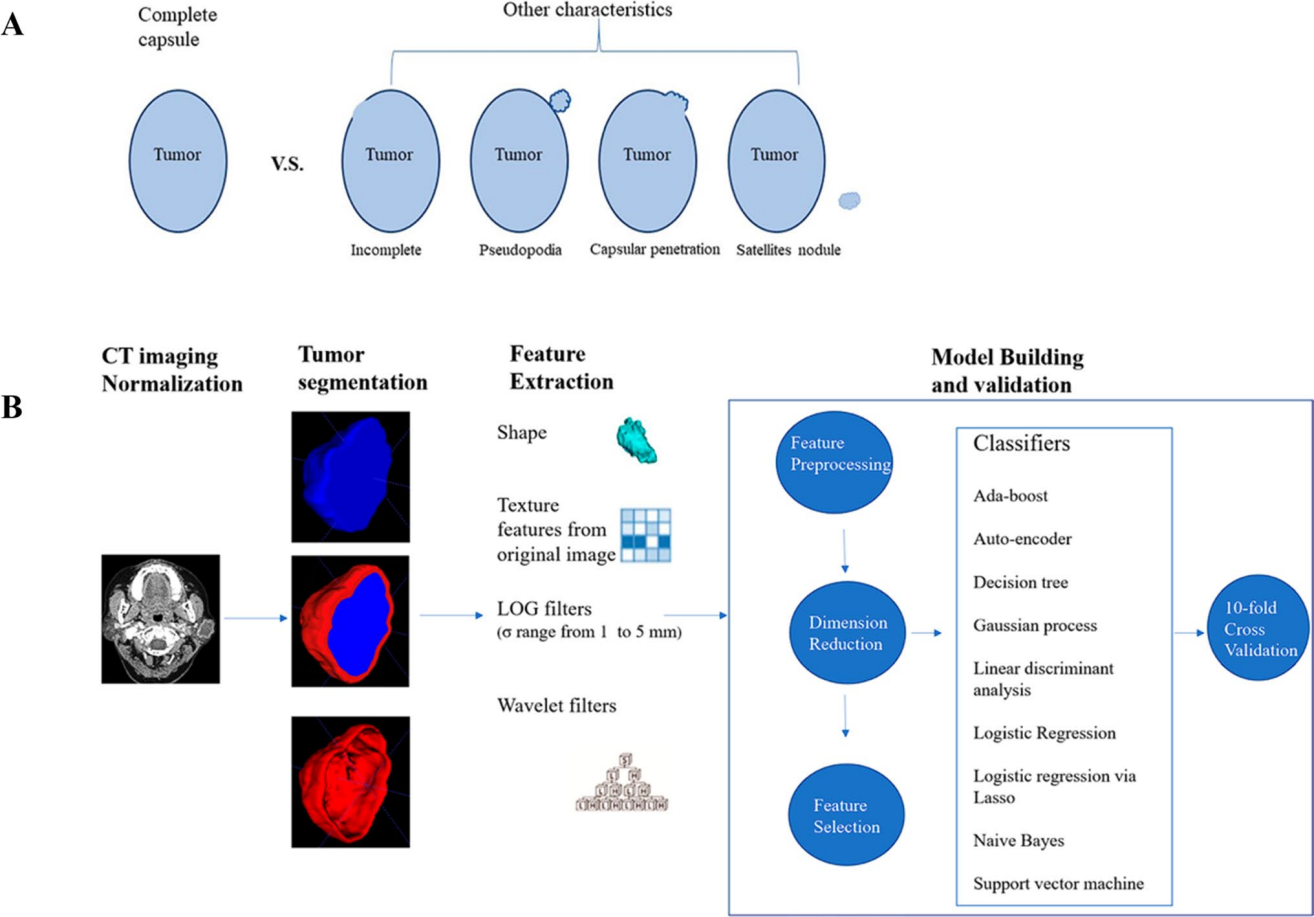
An overview of the current study. **A** All patients were divided into two group based on capsular characteristics. **B** The workflow of this study

We built and validated models using nine machine learning algorithms, including Ada-boost, Auto-encoder, Decision tree, Gaussian process, Linear discriminant analysis, Logistic Regression, Logistic regression via Lasso, Naive Bayes, and Support Vector Machine, which were implemented using Python code and the scikit-learn library. The parameters of the algorithms are list in Additional file 1: Table S3. For each VOI, 180 models were built by combining each machine learning algorithm with 20 different feature sets. The hyperparameters of each model were determined using tenfold cross-validation on the training dataset. To address the imbalance of the training dataset, we employed two methods: the oversampling and Synthetic Minority Over-sampling Technique (SMOTE) [22]. Both methods were used to balance the dataset, ensuring a more accurate representation of the samples during the analysis process. We evaluated the performance of each model using accuracy and receiver operating characteristic (ROC) curves, calculating metrics such as sensitivity, specificity, accuracy, and area under the curve (AUC). The best model was selected by comparing accuracy metrics, and we estimated the 95% confidence interval (CI) using bootstrap with 1000 samples. We conducted feature preprocessing and model exploration with FeAture Explorer Pro (FAE, version 0.5.2) on Python (3.7.6). The overall workflow is summarized in Fig. 2.

### Statistical analysis

The statistical analysis was performed using R software (version 3.6). The Mann–Whitney U-test (clinical duration and maximum diameter) and Student’s t-test (age, it was expressed as mean ± standard deviation) compare continuous variables. Pearson Chi-square and Fisher exact test compare categorical variables. P-value < 0.05 was statistically significant.

## Results

### Patient demographics and clinical parameters

The demographic characteristics showed no significant differences between patients with and without a complete capsule (as shown in Table 1). Although in institution 1, the PA without a complete capsule had a larger diameter (2.38 cm ± 0.70 cm vs. 1.79 cm ± 0.54 cm, P < 0.001), this difference was not significant in institution 2.

**Table 1:** The demographics and clinical parameters of patients with parotid pleomorphic adenoma

Clinical factors	Institution 1			Institution 2		
	PA with complete capsule (n = 92)	PA without complete capsule (n = 74)	*P* value	PA with complete capsule (n = 49)	PA without complete capsule (n = 45)	*P* value
Age (years)	40.99 ± 13.55	44.19 ± 15.19	0.12	47.12 ± 16.06	42.84 ± 14.28	0.17
Sex (male, %)	28, 30.4%	18, 24.3%	0.38	15, 30.6%	15, 33.3%	0.77
Location (right/left)	46/46	42/32	0.38	25/24	25/20	0.66
Tumor margin (well defined/ill defined)	75/17	58/16	0.61	41/8	38/7	0.92
Maximum diameter of tumor (cm)	1.79 ± 0.54	2.38 ± 0.70	< 0.001	2.06 ± 0.65	2.33 ± 0.90	0.07
Calcification (present)	0	3	0.17	2	2	1
Cystic lesion (present)	11	11	0.58	5	4	1

*PA* pleomorphic adenoma

### Comparison of different data balancing techniques

We used the SMOTE and oversampling to make samples balance. They had the similar performance in the training and validation sets. However, the AUC value in the test set was lower when we applied SMOTE. The selection process for the patients is depicted in the flow diagram in Additional file 1: Figure S2.

### Performance of different regions

We compared the AUCs of all the pipelines on the validation dataset. The model using features from the VOI_intra-plus peritumor_ yielded the highest AUC.

**Fig. 3:**
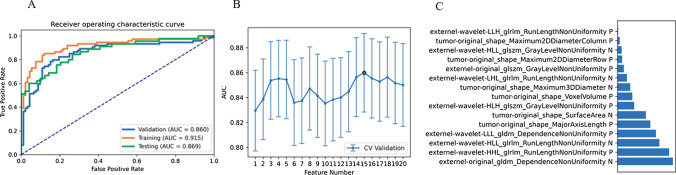
Model performance generated using features from VOI_intra-plus peritumor_. **A** ROC of the top performing model in different datasets. **B** the model based on 15 features can get the highest AUC on the validation data set. **C** The selected features in the model. *AUC* area under the curve, *VOI* region of interest, *ROC* Receiver operating characteristic curves

Among the nine different classifiers, Linear discriminant analysis (LDA) had the best performance (Table 2). LDA was a linear classifier by fitting class conditional densities to the data and using Bayes’ rule. We found that the model based on 15 features can get the highest AUC on the validation data set. The AUC and the accuracy could achieve 0.860 and 0.819, respectively. In this point, The AUC and the accuracy of the model achieve 0.869 and 0.787 on testing data set (as shown in Fig. 3).

**Table 2:** Performance of all algorithm classifications using features from VOI_intra-plus peritumor_

Classifiers	Feature number	Sets	AUC	95% CIs	Accuracy	AUPRC	Sen	Spe	PPV	NPV
LDA	15	Validation	0.860	[0.798–0.921]	0.819	0.842	0.797	0.837	0.797	0.837
		Train	0.915	[0.867–0.962]	0.868	0.917	0.851	0.880	0.851	0.880
SVM	18	Validation	0.857	[0.796–0.919]	0.825	0.827	0.730	0.902	0.857	0.806
		Train	0.903	[0.851–0.954]	0.855	0.911	0.824	0.880	0.847	0.862
LRLasso	15	Validation	0.854	[0.792–0.916]	0.819	0.821	0.770	0.859	0.814	0.823
		Train	0.896	[0.845–0.948]	0.849	0.904	0.730	0.946	0.915	0.813
LR	11	Validation	0.849	[0.786–0.912]	0.807	0.815	0.865	0.761	0.744	0.875
		train	0.882	[0.827–0.936]	0.837	0.878	0.811	0.859	0.822	0.850
AE	2	Validation	0.847	[0.787–0.908]	0.777	0.795	0.851	0.717	0.708	0.857
		Train	0.858	[0.798–0.918]	0.807	0.825	0.838	0.783	0.756	0.857
GP	4	Validation	0.827	[0.760–0.893]	0.783	0.794	0.838	0.739	0.721	0.850
		Train	0.867	[0.811–0.924]	0.831	0.838	0.824	0.837	0.803	0.856
NB	2	Validation	0.822	[0.754–0.891]	0.783	0.784	0.838	0.739	0.721	0.850
		Train	0.862	[0.804–0.919]	0.813	0.826	0.797	0.826	0.787	0.835
AB	8	Validation	0.785	[0.713–0.858]	0.747	0.750	0.797	0.707	0.686	0.813
		Train	0.997	[0.993–1.000]	0.982	0.997	0.973	0.989	0.986	0.979
DT	14	Validation	0.725	[0.656–0.793]	0.723	0.764	0.743	0.707	0.671	0.774
		Train	1	[1.000–1.000]	1	1	1	1	1	1

*AUC* area under curve, *CI* confidence interval, *AUPRC* area under the precision recall curve, *SEN* sensitivity, *SPE* specificity, *PPV* positive predictive value, *NPV* negative predictive value. *Models name* machine learning algorithms_number of features; *LDA* linear discriminant analysis, *SVM* Support Vector Machine, *LRLasso* Logistic Regression via Lasso, *LR* Logistic Regression, *AE* auto-encoder, *GP* Gaussian process, *NB* naive Bayes, *AB* ada-boost, *DT* decision tree

**Fig. 4:**
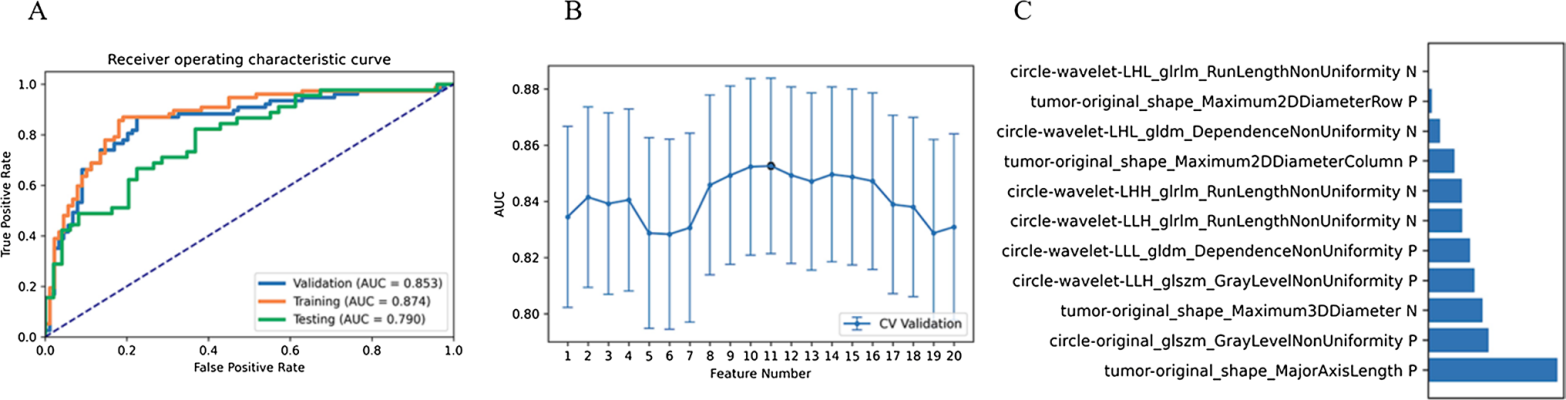
Model performance generated using features from VOI_peritumor_. **A** ROC curves of the best-performing model in different datasets. **B** the model based on 11 features can get the highest AUC on the validation data set. **C** The selected features in the model. *AUC* area under the curve, *VOI* region of interest, *ROC* Receiver operating characteristic curves

The model using features from the VOI_peritumor_ also performed well (when using the Logistic Regression classifier). Out of the nine different classifiers, Logistic Regression (LR) performed the best (as shown in Table 3). LR is a linear classifier that combines all the features. Our analysis revealed that the model based on 11 features achieved the highest AUC on the validation dataset. The AUC and accuracy reached 0.853 and 0.819, respectively. In this point, The AUC and the accuracy of the model achieve 0.790 and 0.723 on testing data set (as shown in Fig. 4).

**Table 3:** The metrics of all algorithm classifications in VOI_peritumor_

Classifiers	Feature number	Sets	AUC	95% CIs	Accuracy	AUPRC	Sen	Spe	PPV	NPV
LR	11	Validation	0.853	[0.792–0.914]	0.819	0.821	0.870	0.775	0.770	0.873
		Train	0.874	[0.818–0.930]	0.837	0.846	0.870	0.809	0.798	0.878
AE	2	Validation	0.850	[0.790–0.910]	0.807	0.821	0.844	0.775	0.765	0.852
		Train	0.862	[0.803–0.920]	0.819	0.828	0.857	0.787	0.777	0.864
LDA	3	Validation	0.849	[0.787–0.910]	0.807	0.811	0.831	0.787	0.771	0.843
		Train	0.862	[0.804–0.921]	0.807	0.835	0.805	0.809	0.785	0.828
LRLasso	4	Validation	0.846	[0.783–0.908]	0.813	0.805	0.844	0.787	0.774	0.854
		Train	0.866	[0.808–0.923]	0.813	0.840	0.818	0.809	0.788	0.837
SVM	11	Validation	0.844	[0.780–0.908]	0.819	0.820	0.805	0.832	0.805	0.832
		Train	0.878	[0.823–0.932]	0.825	0.843	0.831	0.820	0.800	0.849
NB	1	Validation	0.833	[0.769–0.897]	0.781	0.793	0.753	0.809	0.773	0.791
		Train	0.854	[0.795–0.914]	0.801	0.819	0.870	0.742	0.744	0.868
GP	1	Validation	0.829	[0.765–0.894]	0.783	0.777	0.857	0.719	0.725	0.853
		Train	0.855	[0.797–0.914]	0.801	0.838	0.870	0.742	0.744	0.868
AB	7	Validation	0.825	[0.758–0.892]	0.813	0.815	0.766	0.854	0.819	0.809
		Train	0.996	[0.991–1.000]	0.976	0.996	0.974	0.978	0.974	0.978
DT	5	Validation	0.702	[0.632–0.772]	0.705	0.754	0.662	0.742	0.689	0.717
		Train	1.00	[1.000–1.000]	1	1	1	1	1	1

*AUC* area under curve, *CI* confidence interval, *AUPRC* area under the precision recall curve, *SEN* sensitivity, *SPE* specificity, *PPV* positive predictive value, *NPV* negative predictive value. *Models name* machine learning algorithms_number of features; *LDA* linear discriminant analysis, *SVM* Support Vector Machine, *LRLasso* Logistic Regression via Lasso, *LR* Logistic Regression, *AE* auto-encoder, *GP* Gaussian process, *NB* naive Bayes, *AB* ada-boost, *DT* decision tree

**Fig. 5:**
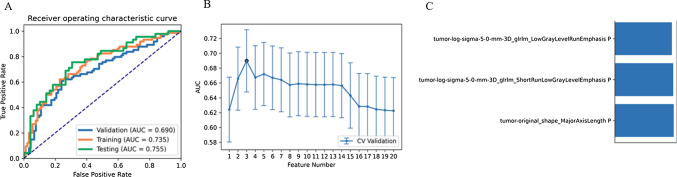
Model performance generated using features from VOI_tumor_. **A** ROC curves of the top performing model in different datasets. **B** the model based on three features can get the highest AUC on the validation data set. **C** The selected features in the model. *AUC* area under the curve, *VOI* region of interest, *ROC* Receiver operating characteristic curves

Regarding the model using VOI_tumor_ features, the pipeline using the Logistic Regression via Lasso (LRLasso) classifier achieved the highest AUC (as shown in Table 4). LRLasso constraint is a linear classifier based on Logistic Regression. The L1 norm is added to the final loss function, and the weights are constrained, resulting in sparse features. Our analysis found that the model based on three features achieved the highest AUC on the validation dataset. The AUC and the accuracy could achieve 0.690 and 0.693, respectively. In this point, The AUC and the accuracy of the model achieve 0.755 and 0.723 on testing data set (as depicted in Fig. 5).

**Table 4:** Performance of all algorithm classifications using features from VOI_tumor_

Classifiers	Feature number	Sets	AUC	95% CIs	Accuracy	AUPRC	Sen	Spe	PPV	NPV
LRLasso	3	Validation	0.690	[0.607–0.772]	0.693	0.626	0.622	0.750	0.667	0.711
		Train	0.735	[0.658–0.812]	0.705	0.680	0.622	0.772	0.687	0.717
LR	3	Validation	0.689	[0.607–0.772]	0.693	0.621	0.635	0.739	0.662	0.716
		Train	0.740	[0.663–0.817]	0.711	0.688	0.635	0.772	0.691	0.725
LDA	3	Validation	0.682	[0.598–0.765]	0.681	0.638	0.595	0.750	0.657	0.697
		Train	0.740	[0.663–0.817]	0.711	0.685	0.635	0.772	0.691	0.725
SVM	5	Validation	0.681	[0.598–0.763]	0.663	0.611	0.676	0.652	0.610	0.714
		Train	0.744	[0.668–0.821]	0.693	0.694	0.784	0.620	0.624	0.781
AE	19	Validation	0.679	[0.597–0.762]	0.651	0.605	0.757	0.565	0.583	0.743
		Train	0.717	[0.638–0.797]	0.717	0.673	0.608	0.804	0.714	0.718
NB	3	Validation	0.669	[0.585–0.754]	0.675	0.594	0.595	0.739	0.647	0.694
		Train	0.731	[0.653–0.808]	0.705	0.673	0.662	0.739	0.671	0.731
GP	3	Validation	0.646	[0.558–0.734]	0.657	0.618	0.649	0.663	0.608	0.701
		Train	0.759	[0.686–0.833]	0.717	0.729	0.649	0.772	0.696	0.732
AB	1	Validation	0.582	[0.493–0.671]	0.608	0.501	0.473	0.717	0.574	0.629
		Train	0.873	[0.822–0.924]	0.795	0.866	0.811	0.783	0.750	0.837
DT	3	Validation	0.546	[0.470–0.623]	0.548	0.616	0.527	0.565	0.494	0.598
		Train	1	[1.000–1.000]	1	1	1	1	1	1

*AUC* area under curve, *CI* confidence interval, *AUPRC* area under the precision recall curve, *SEN* sensitivity, *SPE* specificity, *PPV* positive predictive value, *NPV* negative predictive value. *Models name* machine learning algorithms_number of features; *LDA* linear discriminant analysis, *SVM* Support Vector Machine, *LRLasso* Logistic Regression via Lasso, *LR* Logistic Regression, *AE* auto-encoder, *GP* Gaussian process, *NB* naive Bayes, *AB* ada-boost, *DT* decision tree

### Comparison of AUCs

**Fig. 6:**
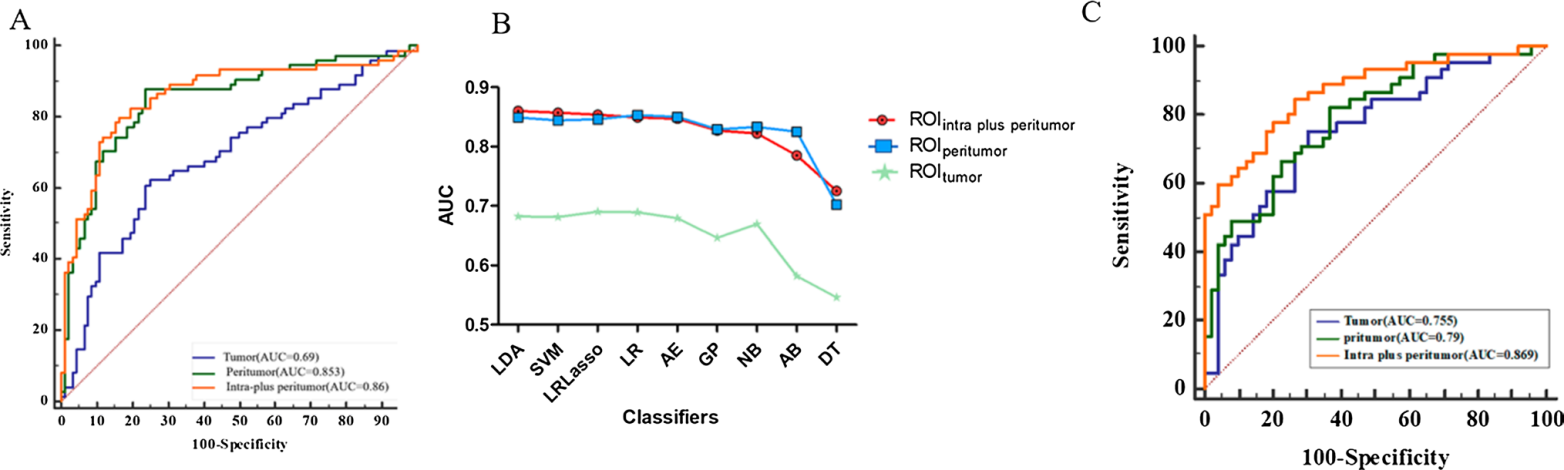
Comparison of the performance of Models from VOI_tumor_, VOI_peritumor_, and VOI_intra-plus peritumor_. **A** ROC curves of the top performing models in the tenfold cross-validation. **B** AUC values of the nine different machine learning algorithm-based models in the tenfold cross-validation. **C** ROC curves of the top performing models in the test set. *AUC* area under the curve, *VOI* region of interest, *ROC* Receiver operating characteristic curves, *LDA* Linear discriminant analysis, *SVM* Support vector machine, *LRlasson* Logistic Regression via Lasso, *LR* Logistic Regression, *AE* Auto-encoder, *GP* Gaussian process, *NB* Naive Bayes, *AB* Ada-boost, *DT* Decision tree

The Delong test showed that the AUCs were significantly different between VOI_intra-plus peritumor_ and VOI_tumor_ (AUC: 0.86 vs. 0.69; difference between areas = 0.17, P = 0.0002), VOI_peritumor_ and VOI_tumor_ (AUC: 0.853 vs. 0.69; difference between areas = 0.16, P = 0.0007) in the validation set. In the test set, the AUCs were significantly different between VOI_intra-plus peritumor_ and VOI_tumor_ (AUC: 0.869 vs. 0.755; difference between areas = 0.11, P = 0.018), as depicted in Fig. 6.

## Discussion

In this study, we aimed to develop and validate radiomics models based on CT images to differentiate between PA with and without complete capsule. Features were extracted from three VOIs: VOI_tumor_, VOI_intra-plus peritumor_, and VOI_peritumor_. The latter two VOIs contained the peritumoral region. Our results indicate that the use of intra-plus peritumoral radiomics features may provide a non-invasive method for determining the complete capsule status, which is important for surgical planning and treatment decisions.

The peritumoral region is the area surrounding the primary tumor and can provide important information about the tumor’s microenvironment, including its interactions with surrounding tissues and its potential for malignant behavior. The value of peritumoral radiomics features has been demonstrated in previous studies, showcasing their potential in characterizing tumor behavior, predicting treatment response, and assessing the risk of recurrence and metastasis [23–27]. However, their application in parotid adenomas (PA) has not yet been explored. Various studies have explored different definitions of the peritumoral region, including distances ranging from 2 to 12 mm for gastrointestinal stromal tumor, 3 mm to 15 mm for breast cancer, and 5 mm to 30 mm for lung nodules. The results of these studies suggest that the peritumoral region closest to the lesion typically yields the best performance in terms of predictive accuracy [23, 25, 27]. Besides, a specific fixed distance (3 mm) was also defined as the peritumoral region for lung cancer [24].

In the current study, we defined 2 mm around the tumor as the peritumoral region and did not investigate further distance. There are several reasons. First, the capsular characteristics occur at the margin of the tumor, and even satellite nodules are mostly found within 2 mm from the central mass [19]. Thus, making 2 mm around the tumor sufficient for the evaluation of capsular characteristics. Additionally, Wu et al. [27] found that the radiomics model for laryngeal carcinoma will collapse when the distance was farther than 4.5 mm, which confirms that a larger peritumoral region does not necessarily lead to better performance and that the optimal distance should be set based on pathological characteristics. Secondly, the parotid gland is smaller in size compared to the stomach, lung, or breast and therefore a shorter distance of extension is needed to avoid going beyond the border of the parotid.

Our study found that the radiomics features from VOI_intra-plus peritumor_ showed the best performance in predicting capsular characteristics of parotid PA. The most important features were those related to heterogeneity and complexity of the texture, such as Dependence Non-Uniformity, Run Length Non-Uniformity, and Gray Level Non-Uniformity. These features measure non-uniformity in image intensity [28–30]. Differences in capsular characteristics may lead to variations in the micro-structures of the peritumoral regions. In patients with a complete capsule, the peritumoral region is composed exclusively of parotid tissue. In contrast, those without a complete capsule have a peritumoral region containing both parotid and tumor tissues, resulting in increased heterogeneity. Furthermore, shape-based features, such as maximum 2D diameter, maximum 3D diameter, and major axis length, were also significant in the developed radiomics model. This is consistent with prior studies that have identified a relationship between tumor size and capsular integrity, with larger tumors being more likely to have an incomplete encapsulation [31].

Our study has some limitations. Firstly, a significant number of patients were lost to follow-up, so the actual recurrence rate could not be determined. Additionally, MRI images may provide more valuable information and have less artifact compared to CT images. It may be beneficial to explore the potential of MRI-based radiomics features in future studies. Lastly, our study population was limited to patients from two institutions, and the sample size was not large, so we still need to generalize the results to other populations. Furthermore, alternative advanced methods and semi-automatic workflows, such as the one offered by matRadiomics [32], could potentially offer better performance, and further improve the efficiency of the radiomics workflow in a routine clinical setting. This would allow for a more comprehensive assessment of the tumor’s microenvironment and its interactions with surrounding tissues, leading to more accurate predictive models.

In conclusion, our study demonstrated the potential of using peritumoral radiomics features for accurately detecting capsular morphological features of parotid PA. Despite some limitations, the results provide promising evidence for the application of this method in advancing precise treatment for patients with parotid PA. However, further systematic studies, including comparisons with MRI-based radiomics features, are necessary to fully validate the use of peritumoral radiomics in clinical practice.

## Supplementary Information

**Additional file 1.** Figures S1, S2; CT protocols; Tables S1–S3.

## Data Availability

Data is available on request from the authors.

## Abbreviations

AE: Auto-encoder
AB: Ada-boost
AUC: Area under the curve
AUPRC: Area under the precision recall curve
CT: Computed tomography
CI: Confidence interval
DT: Decision tree
GLCM: Gray-level co-occurrence matrix
GLRLM: Gray level run length matrix
GLSZM: Gray level size zone matrix
GLDM: Gray level dependence matrix
GP: Gaussian process
KW: Kruskal Wallis
LoG: Laplacian of Gaussian
LDA: Linear discriminant analysis
LRLasso: Logistic regression via Lasso
LR: Logistic Regression
NGTDM: Neighboring gray-tone difference matrix
NB: Naive Bayes
PA: Pleomorphic adenoma
PCC: Pearson correlation coefficient
ROC: Receiver operator characteristic curve
SMOTE: Synthetic Minority Over-sampling Technique
SVM: Support Vector Machine
VOI: Volume of interest

## Code availability

All data code used during the study are available online.
