# Supplementary material for: CT based intratumor and peritumoral radiomics for differentiating complete from incomplete capsular characteristics of parotid pleomorphic adenoma: a two-center study
Source: Discov Oncol. 2023 May 22;14:76. doi: 10.1007/s12672-023-00665-8 (PMC10203084; doi:10.1007/s12672-023-00665-8)
Supplement: Supplementary file 1 — Additional file 1. Figures S1, S2; CT protocols; Tables S1–S3. [file 12672_2023_665_MOESM1_ESM.docx]

**Additional file: Figure S1; Figure S2 ; CT protocols; Table S1; Table S2**

**Figure S1. Flow chart depicting patient’s enrollment process**


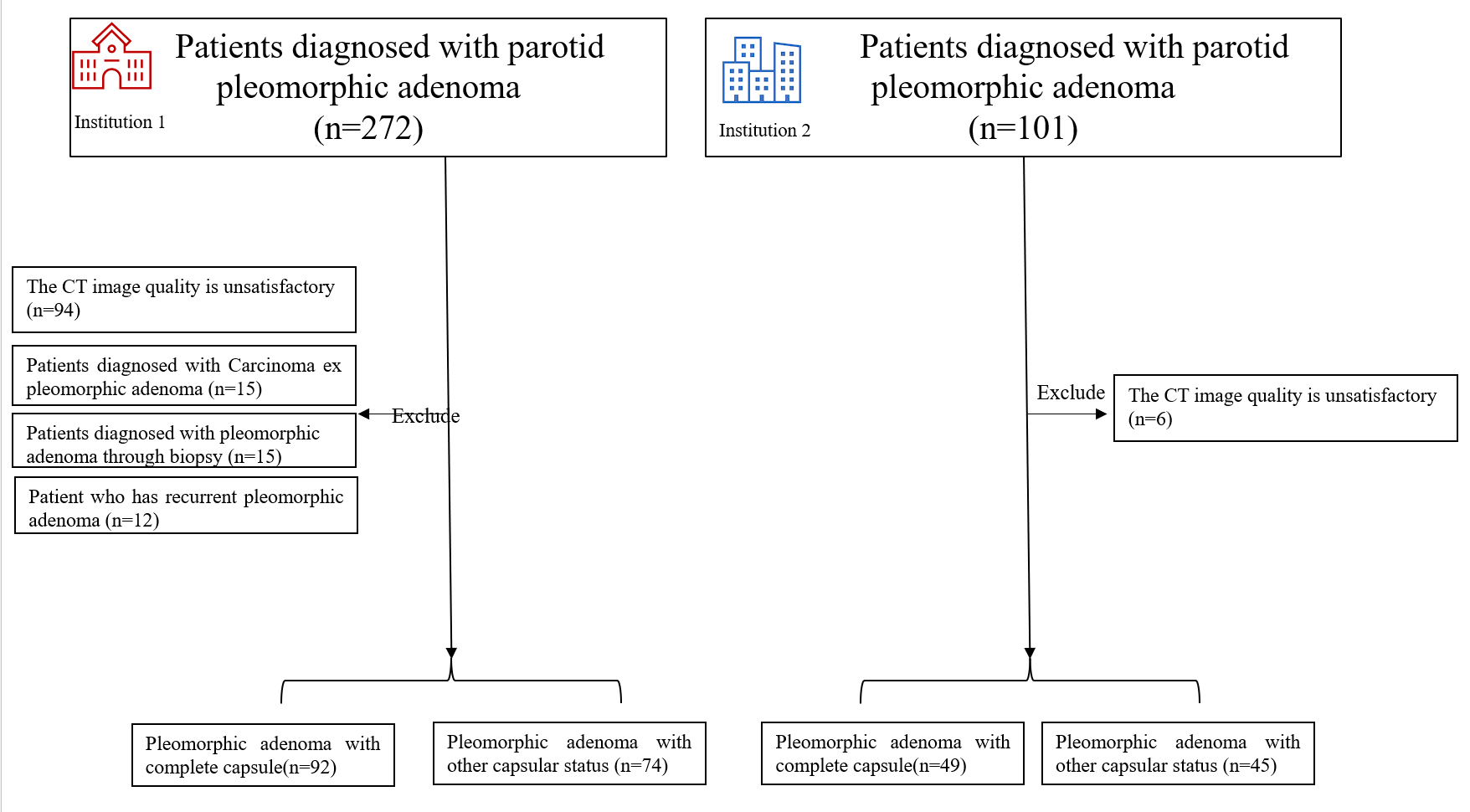


**Note: CT=Computed tomography**

**Figure S2. The performance of SMOTE (A) and Oversampling (B) in the training and validation sets.**

**
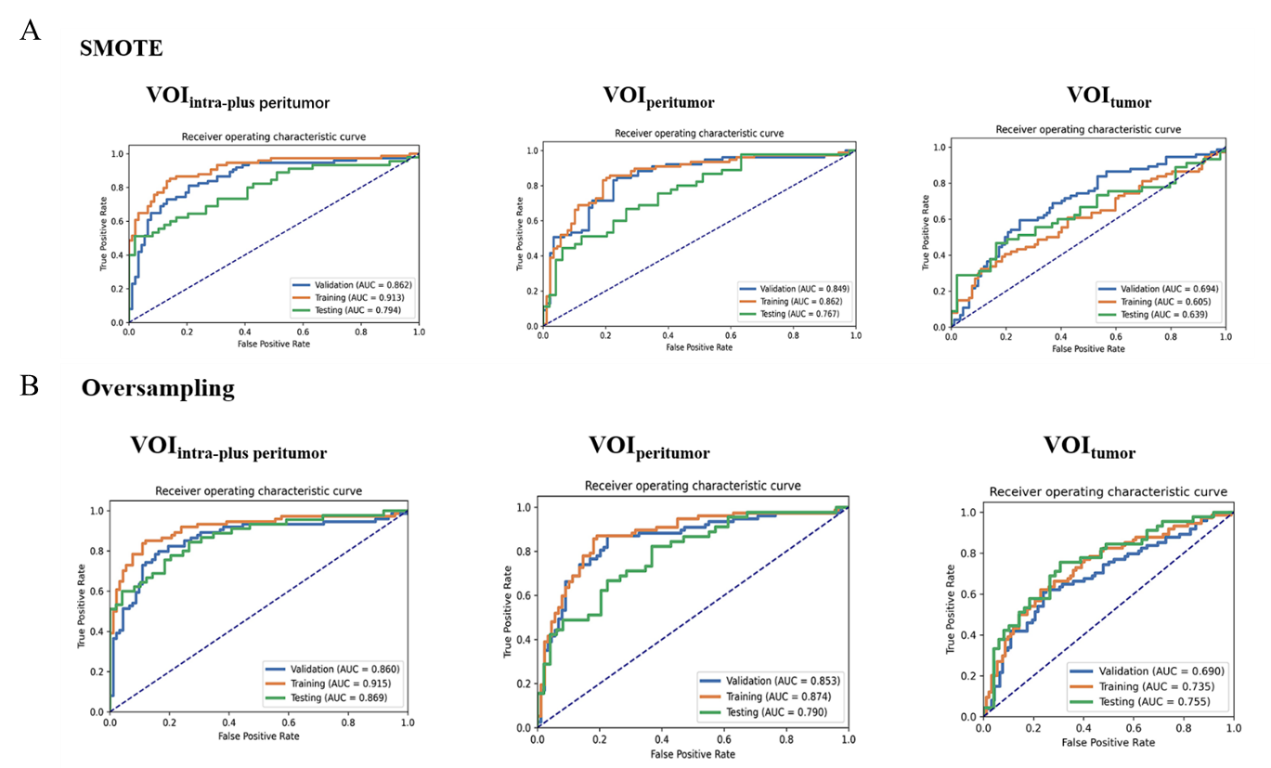
**

**Note:** **SMOTE =** **Synthetic Minority Over-sampling Technique**

**CT protocols of two centers**

The patients underwent CT on 3 scanners in institution 1, including SOMATOM Definition Flash (Siemens, Germany); SOMATOM Definition Force (Siemens, Germany); Revolution CT (GE MEDICAL SYSTEMS, Waukesha, WI, USA). The patients underwent CT on 3 scanners in institution 2, Discovery CT750 HD (GE MEDICAL SYSTEMS, Waukesha, WI, USA); SOMATOM Definition Flash (Siemens, Germany); SOMATOM Definition Force (Siemens, Germany). After injection of infused 1.5 ml/kg body weight iodinated contrast agent, the contrast-enhanced CT images are obtained. Arterial phase scan is acquired in 30-35 seconds after intravenous.The contrast agent is injected intravenously at a flow rate of 3.0 ml/s. Acquisition parameters: tube voltage is 120KVp; automatic tube current modulation is applied; section thickness is 5 mm; section interval is 5 mm ; tube rotation time is 0.5-1.0s; spiral pitch factor is 0.7-1.0; image matrix (slice) is 512 × 512 pixels.

Table S1 The list of all extracted textural features.

|  | |
| --- | --- |
| Matrixes | Features |
| Shape | 1. Elongation 2. Flatness 3. Least Axis Length 4. Major Axis Length 5. Maximum 2D Diameter Column 6. Maximum 2D Diameter Row 7. Maximum 2D Diameter Slice 8. Maximum 3D Diameter 9. Mesh Volume 10. Minor Axis Length 11. Sphericity 12. Surface Area 13. Surface Volume Ratio 14. Voxel Volume |
| First Order | 1. 10 Percentile 2. 90 Percentile 3. Energy 4. Entropy 5. Interquartile Range 6. Kurtosis 7. Maximum 8. Mean Absolute Deviation 9. Mean 10. Median 11. Minimum 12. Range 13. Robust Mean Absolute Deviation 14. Root Mean Squared 15. Skewness 16. Total Energy 17. Uniformity 18. Variance |
| Gray Level Co-occurrence Matrix (GLCM) | 1. Autocorrelation 2. Joint Average 3. Cluster Prominence 4. Cluster Shade 5. Cluster Tendency 6. Contrast 7. Correlation 8. Difference Average 9. Difference Entropy 10. Difference Variance 11. Joint Energy 12. Joint Entropy 13. Informational Measure of Correlation (IMC) 1 14. Informational Measure of Correlation (IMC) 2 15. Inverse Difference Moment (IDM) 16. Inverse Difference Moment Normalized (IDMN) 17. Inverse Difference (ID) 18. Inverse Difference Normalized (IDN) 19. Inverse Variance 20. Maximum Probability 21. Sum Entropy 22. Sum Squares |
| Gray Level Run Length Matrix (GLRLM) | 1. Short Run Emphasis (SRE) 2. Long Run Emphasis (LRE) 3. Gray Level Non-Uniformity (GLN) 4. Gray Level Non-Uniformity Normalized (GLNN) 5. Run Length Non-Uniformity (RLN) 6. Run Length Non-Uniformity Normalized (RLNN) 7. Run Percentage (RP) 8. Gray Level Variance (GLV) 9. Run Variance (RV) 10. Run Entropy (RE) 11. Low Gray Level Run Emphasis (LGLRE) 12. High Gray Level Run Emphasis (HGLRE) 13. Short Run Low Gray Level Emphasis (SRLGLE) 14. Short Run High Gray Level Emphasis (SRHGLE) 15. Long Run Low Gray Level Emphasis (LRLGLE) 16. Long Run High Gray Level Emphasis (LRHGLE) |
| Gray Level Size Zone Matrix (GLSZM) | 1. Small Area Emphasis (SAE) 2. Large Area Emphasis (LAE) 3. Gray Level Non-Uniformity (GLN) 4. Gray Level Non-Uniformity Normalized (GLNN) 5. Size-Zone Non-Uniformity (SZN) 6. Size-Zone Non-Uniformity Normalized (SZNN) 7. Zone Percentage (ZP) 8. Gray Level Variance (GLV) 9. Zone Variance (ZV) 10. Zone Entropy (ZE) 11. Low Gray Level Zone Emphasis (LGLZE) 12. High Gray Level Zone Emphasis (HGLZE) 13. Small Area Low Gray Level Emphasis (SALGLE) 14. Small Area High Gray Level Emphasis (SAHGLE) 15. Large Area Low Gray Level Emphasis (LALGLE) 16. Large Area High Gray Level Emphasis (LAHGLE) |
| Neigbouring Gray Tone Difference Matrix (NGTDM) | 1. Coarseness 2. Contrast 3. Busyness 4. Complexity 5. Strength |
| Gray Level Dependence Matrix (GLDM) | 1. Small Dependence Emphasis (SDE) 2. Large Dependence Emphasis (LDE) 3. Gray Level Non-Uniformity (GLN) 4. Dependence Non-Uniformity (DN) 5. Dependence Non-Uniformity Normalized (DNN) 6. Gray Level Variance (GLV) 7. Dependence Variance (DV) 8. Dependence Entropy (DE) 9. Low Gray Level Emphasis (LGLE) 10. High Gray Level Emphasis (HGLE) 11. Small Dependence Low Gray Level Emphasis (SDLGLE) 12. Small Dependence High Gray Level Emphasis (SDHGLE) 13. Large Dependence Low Gray Level Emphasis (LDLGLE) 14. Large Dependence High Gray Level Emphasis (LDHGLE) |
| Exact definition and detailed description of each feature can be found on https://ibsi.readthedocs.io or https://pyradiomics.readthedocs.io | |

# Table S2. The parameters used in CT image pre-processing and feature extraction

| Parameter | Setting |
| --- | --- |
| Intensity normalization | Z-score normalization |
| Intensity Discretization | Fixed Bin Width: 25 HU |
| Spatial Resampling | Voxel Size: 1x1x1 mm³ |
| Wavelet Decomposition | Coif 1 |
| Wavelet Levels | Single-level decomposition. It decomposes the original image into eight wavelet-filtered images, representing various combinations of high-pass (H) and low-pass (L) filters in the x, y, and z directions: LLH, LHL, LHH, HLL, HLH, HHL, LLL, and HHH. |

**Table S3. The parameters of the algorithms**

| **Algorithms** | **Parameters** |
| --- | --- |
| Ada-boost | base_estimator=None, *, n_estimators=50, learning_rate=1.0, algorithm=‘SAMME.R’, random_state=None |
| Auto-encoder | Hidden_layer_sizes=(100), activation=‘relu’, *, solver=‘adam’, alpha=0.0001, batch_size=‘auto’, learning_rate=‘constant’,  learning_rate_init=0.001, power_t=0.5, max_iter=200, shufe=True, random_state=None, tol=0.0001, verbose=False,  warm_start=False, momentum=0.9, nesterovs_momentum=True, early_stopping=False, validation_fraction=0.1,  beta_1=0.9, beta_2=0.999, epsilon=1e-08, n_iter_no_change=10, max_fun=15,000 |
| Decision tree | criterion=‘gini’, splitter=‘best’, max_depth=None, min_samples_split=2, min_samples_leaf=1, min_weight_fraction_  leaf=0.0, max_features=None, random_state=None, max_leaf_nodes=None, min_impurity_decrease=0.0, min_impurity_  split=None, class_weight=None, ccp_alpha=0.0 |
| Gaussian process | kernel=None, *, optimizer=‘fmin_l_bfgs_b’, n_restarts_optimizer=0, max_iter_predict=100, warm_start=False, copy_X_  train=True, random_state=None, multi_class=‘one_vs_rest’, n_jobs=None |
| Linear discriminant analysis | solver=‘svd’, shrinkage=None, priors=None, n_components=None, store_covariance=False, tol=0.0001 |
| Logistic Regression | penalty=‘l2’, *, dual=False, tol=0.0001, C=1.0, ft_intercept=True, intercept_scaling=1, class_weight=None, random_state=None, solver=‘lbfgs’, max_iter=100, multi_class=‘auto’, verbose=0, warm_start=False, n_jobs=None, l1_  ratio=None |
| Logistic regression via Lasso | alpha=1.0, *, ft_intercept=True, normalize=False, precompute=False, copy_X=True, max_iter=1000, tol=0.0001, warm_  start=False, positive=False, random_state=None, selection=‘cyclic’ |
| Naive Bayes | alpha=1.0, binarize=0.0, ft_prior=True, class_prior=None |
| Support vector machine | C=1.0, kernel=‘rbf’, degree=3, gamma=‘scale’, coef0=0.0, shrinking=True, probability=False, tol=0.001, cache_size=200,  class_weight=None,mverbose=False, max_iter=—1, decision_function_shape=‘ovr’, break_ties=False, random_state=None |
